# A Subset of Mouse Colonic Goblet Cells Expresses the Bitter Taste Receptor Tas2r131

**DOI:** 10.1371/journal.pone.0082820

**Published:** 2013-12-18

**Authors:** Simone Prandi, Marta Bromke, Sandra Hübner, Anja Voigt, Ulrich Boehm, Wolfgang Meyerhof, Maik Behrens

**Affiliations:** 1 German Institute of Human Nutrition Potsdam-Rehbruecke, Dept. Molecular Genetics, Nuthetal, Germany; 2 University of Saarland, School of Medicine, Department of Pharmacology and Toxicology, Homburg, Germany; Barnard College, Columbia University, United States of America

## Abstract

The concept that gut nutrient sensing involves taste receptors has been fueled by recent reports associating the expression of taste receptors and taste-associated signaling molecules in the gut and in gut-derived cell lines with physiological responses induced by known taste stimuli. However, for bitter taste receptors (Tas2rs), direct evidence for their functional role in gut physiology is scarce and their cellular expression pattern remained unknown. We therefore investigated Tas2r expression in mice. RT-PCR experiments assessed the presence of mRNA for Tas2rs and taste signaling molecules in the gut. A gene-targeted mouse strain was established to visualize and identify cell types expressing the bitter receptor Tas2r131. Messenger RNA for various Tas2rs and taste signaling molecules were detected by RT-PCR in the gut. Using our knock-in mouse strain we demonstrate that a subset of colonic goblet cells express Tas2r131. Cells that express this receptor are absent in the upper gut and do not correspond to enteroendocrine and brush cells. Expression in colonic goblet cells is consistent with a role of Tas2rs in defense mechanisms against potentially harmful xenobiotics.

## Introduction

In recent years numerous studies addressed the presence of taste receptors and taste-associated signaling components outside the gustatory system. One of these sites is the gastrointestinal (GI) tract (for review see [1]). The discovery of taste-associated molecules in the gut has led to the hypothesis that taste receptors are part of a chemosensory mechanism in gut epithelium, able to recognize nutrients and chemicals entering the digestive tract, and trigger diverse physiological processes [2]. For example, the expression of taste-signaling components and of Tas1r2 as well as Tas1r3, i.e., the sweet receptor subunits, has been associated with specific cell types by histological methods and with physiological functions such as glucose uptake and the regulation of blood glucose levels [3], [4]. The identified cell types include enteroendocrine and brush cells [3], [5]–[7].

The TAS2R family comprises of ∼25 members in humans and over 30 members in rodents [8]. This limited set of receptors recognizes thousands of different bitter substances [9]. Expression of bitter taste receptors (named TAS2Rs (human) or Tas2rs (rodents)) has also been reported in the gut but is mostly based on RT-PCR results and studies of GI-tract derived cell lines (e.g. [10]–[14]). Thus, the cell types in the gut which express TAS2Rs remain unidentified making it impossible to predict reliably their physiological functions. Nevertheless, bitter substance-evoked gut responses have been observed. For example, bitter tasting 6-*n*-propyl-2-thiouracil induces increased anion secretion in large intestine [11], intragastric infusion of denatonium benzoate delays gastric emptying in rats [15] and bitter agonists regulate ghrelin secretion in mice [16]. Some bitter tastants evoke Ca^2+^ responses and release of hormones such as cholecystokinin or glucagon-like peptide-1 (GLP-1) in gut-derived endocrine cell lines expressing TAS2Rs (e.g. [10], [12]–[14]). However, data are lacking demonstrating a causative link between the bitter-stimulus induced responses and the expressed TAS2Rs. Firstly, neither have knock-out mice been investigated nor have RNAi-knock-down experiments targeting directly TAS2Rs been reported. Secondly, the concentrations of bitter substances used in recent studies do not match those required for TAS2R activation in cell-based assays. Thirdly, the bitter compound-evoked responses do not correspond to the TAS2Rs found to be expressed. For example, phenylthiocarbamide (PTC) evoked calcium responses in NCI-H716 cells [12] at concentrations that were more than 1000-fold higher than those required to activate the single cognate TAS2R, i.e. TAS2R38 [17]. Moreover, these cells express a defective TAS2R38 variant [12] that fails to respond to agonist stimulation [17]. Thus, the PTC-response occurs independently of TAS2R action. Fourthly, it is known that, besides their cognate TAS2Rs, bitter substances have other cellular targets, well known examples being the inhibition of phosphodiesterases by caffeine [18], and of ATP-sensitive potassium channels by denatonium as well as the binding of hop humulones to a nuclear receptor (see [1] and references therein).

In this study, we set out to elucidate the localization and assumed role of TAS2Rs in the GI-tract. We focused on mouse tissues to examine Tas2r expression *in situ*. Finally, we analyzed GI tissues of a new gene-targeted mouse strain carrying a recombinant *Tas2r131* allele to identify and characterize Tas2r-expressing cells.

## Materials and Methods

### Ethics statement

Animal care and experimental procedures were performed in accordance with the guidelines of and approved by the animal welfare committee of the University of Hamburg and the Ministry of Environment, Health and Consumer Protection of the state of Brandenburg (permit number 23-2347-A-1-1-2010).*Animals-* Wild type C57BL/6 as well as gene-targeted mice were used in all studies. Mice were kept under standard light/dark cycle with water and food *ad libitum*.

A description of the generation of Tas2r131*^F/MBLF/M-IRES-Cre^*mice (short: Tas2r131^BLiC^) has been published [19]. Briefly, both 5′- and 3′-flanking fragments of the *Tas2r131* coding region were combined in a construct consisting of a modified version of barley lectin, an internal ribosome entry site, Cre recombinase, and a neomycin resistance cassette flanked by FRT sites 3′ of the Cre recombinase sequence. After successful transfer into embryonic stem (ES) cells of a 129/Sv mouse strain, ES cell clones were used to generate Tas2r131*^BLiC^* ^neo+^-mice. ES cells were injected into blastocysts (C57BL/6), implanted into (C57BL/6 x DBA) F1 foster mothers and subsequently, male chimeras were backcrossed with C57BL/6 females. Breeding with FLP recombinase deleter mouse strain [20] resulted in Tas2r131*^BLiC^*-mice. To create a reporter strain expressing tandem dimer Red Fluorescent Protein (tdRFP) in a Cre recombinase-dependent manner, Tas2r131*^BLiC^* and Rosa26:stopp^floxed^tdRFP [21] mice (short: ROSA26^tdRFP^) were crossed. The resulting heterozygous Tas2r131^+/*BLiC*^/ROSA26^+/tdRFP^ mice and littermates carrying *Tas2r131* wild type allele were used in this study.

### Tissue collection and preparation

For *in situ* hybridization and immunohistochemistry, tissues were obtained from transcardially perfused animals. Perfusion of deeply anesthetized animals, postfixation, cryoprotection and freezing of tissues were done as before [22]. Before freezing, intestinal samples (jejunum, 4–10 cm post-pylorus; colon 0–4 cm from rectum) were rolled along the longitudinal axis. Cryostat sections of vallate papillae (VP) and intestines were prepared as reported previously [23]. For RNA isolation tissues were obtained from non-perfused animals sacrificed by cervical dislocation and immediately shock-frozen in liquid nitrogen.

### Reverse-transcription PCR

Total RNA was extracted from mouse tissues using TRIzol reagent (Invitrogen). Poly(A)^+^ RNA was isolated with MicroPoly(A)Purist Kit (Applied Biosystems, Darmstadt, Germany). DNAse I (Invitrogen) digestion and cDNA synthesis were performed as before [23]. For each sample, a negative control omitting reverse transcriptase was prepared. PCR was performed on cDNA corresponding to ∼25 ng poly(A)^+^ RNA. Reaction conditions: 5 min 94°C; 40 cycles (30 cycles for glyceraldehyde-3-phosphate dehydrogenase) of 90 sec at specified annealing temperature, 90 sec 72°C, 30 sec 94°C; 5 min at specified annealing temperature and 10 min 72°C. Primer sequences for analytical RT-PCR experiments, length of amplicons and annealing temperatures see Table S1.

For quantitative RT- PCR total RNA was extracted from mouse GI tissues using TRIzol reagent. After DNAse I digestion reverse transcription was realized using Superscript III (Invitrogen) and random primers (Invitrogen). Reverse transcriptase was omitted in samples serving as negative controls (-RT). Gene specific primers combined with *TaqMan* probes were used to amplify cDNAs (see table S2 for a list of oligonucleotides). TaqMan assays were designed using *Primer Express 3.0* software (Applied Biosystems) and ordered at *Eurofins MWG Operon*. Housekeeping gene expression (β-actin-sequence, see table S2) served as internal control. Real-time PCR was performed using *7500 Fast Real-Time PCR System* (Applied Biosystems). For PCR template cDNA corresponding to 25 ng total RNA was included in a final sample volume of 10 µl. In addition, the sample contained 0.5 µM probe, 1.25 µM of each forward and reverse primer, and 1x *TaqMan® Gene Expression Master Mix* (Applied Biosystems). Cycling parameters were as follows: 50°C for 2 min for preincubation, and 95°C for 10 min for polymerase activation and initial denaturation; followed by 40 cycles of 15 sec at 95°C for denaturation, and 1 min at 60°C for annealing and elongation. Each cDNA (+RT) sample was tested in triplicates; -RT and water instead of template were used as controls for unspecific expression for each gene. Threshold cycle (C_T_) values were reported in *7500 Software v2.0.1* software (Applied Biosystems) using experimental property *Quantitation-Comparative CT (ΔΔCT)*. Raw data were further analyzed using *Microsoft Excel* software. Mean values of C_T_ triplets were obtained (single values differing ≥1 C_T_ were excluded). 2^−ΔCT^ values were calculated using mean C_T_ of β-actin as reference (ΔC_T_ = C_T_ target − C_T_ reference).

### Immunohistochemistry

For the detection of Tas2r138, α-gustducin, PLCβ2, chromogranin A, GLP-1, villin, mucin-2, cytokeratin 18 tissue sections of C57BL/6 and Tas2r131^+/*BLiC*^/ROSA^+/tdRFP^ mice were used. For detailed staining procedures see Materials and Methods S1.

### In situ hybridization

The preparation of digoxygenin-labeled riboprobes and *in situ* hybridization were performed as described before [23]. Briefly, coding sequences of Tas2rs detected by RT-PCR in mouse GI tissues, an α-gustducin fragment and a Cre recombinase fragment were cloned into pBluescript I KS (Stratagene). *In vitro* transcription reactions were performed to produce probes for α-gustducin (733 bp), Cre recombinase (410 bp), and 5 Tas2rs: Tas2r108 (894 bp), Tas2r138 (455 bp), Tas2r118 (1000 bp), Tas2r131 (524 bp), and Tas2r119 (1002 bp), applied together as a mixture. Hybridization was done as described previously [23] with an additional proteinase K digestion step after initial washing and permeabilization (5 min in PBS with 0.6 units proteinase K, Roche). Digestion was stopped with 0.2% glycine solution and two washes with PBS. Postfixation with 4% paraformaldehyde was performed prior to acetylation. Finally, slides were analyzed with a Zeiss Axioplan microscope (Zeiss) connected to a CCD camera (RT slider; Diagnostic Instruments).

## Results

### Tas2rs and taste-related signaling proteins are expressed in mouse GI tissues

**Figure 1:**
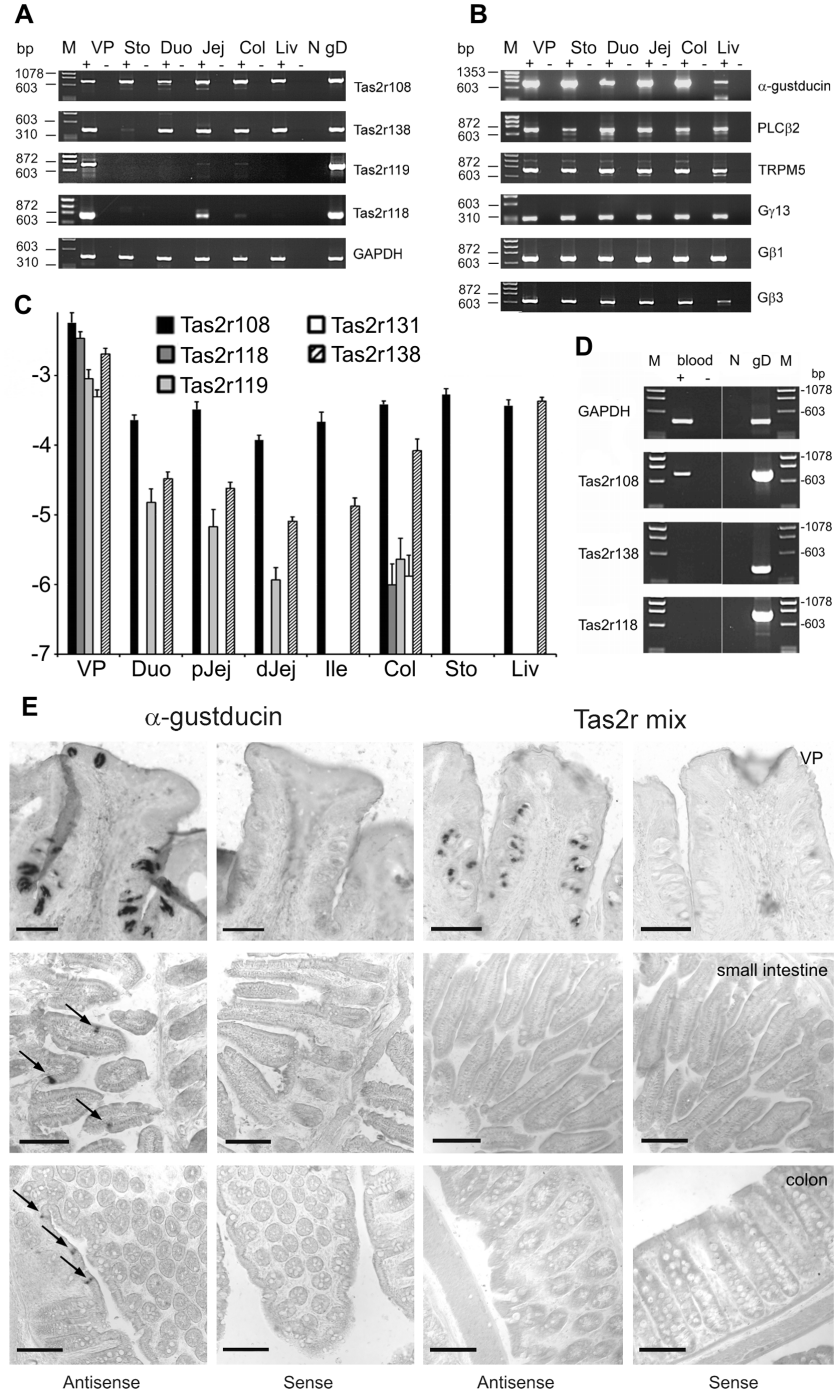
Expression analysis of Tas2rs and taste signaling cascade elements in mouse tissues. (A) and (B) PCR analyses of cDNA from vallate papillae (VP), stomach (Sto), duodenum (Duo), jejunum (Jej), colon (Col) and liver (Liv). (A) PCR products obtained from cDNA of mouse GI tissues (+) and -RT controls (−) with primers specific for Tas2r108, -r138, -r119 and -r118. GAPDH was amplified as quality control, mouse genomic DNA served as positive control (gD). N, negative control (H_2_O). (B) PCR analysis of α-gustducin, PLCβ2, TRPM5, Gγ13, Gβ1 and Gβ3 expression in mouse GI tract. M - Molecular weight standard (ΦX174 DNA/HaeIII). (C) Quantitative RT-PCR of gustatory and gastrointestinal tissues. cDNA of vallate papillae (VP), duodenum (Duo), proximal (pJej) and distal (dJej) jejunum, ileum (Ile), colon (Col), stomach (Sto) and liver (Liv) was subjected to quantitative real-time PCR analyses using primers specific for Tas2r108 (black bars), -r118 (dark gray bars), -r119 (light gray bars), -r131 (white bars), and –r138 (hatched bars). Y-axis  =  logarithm of the expression levels relative to β-actin (mean log(2^−ΔCT^) ±SE, n≥3). (D) Analysis of Tas2r gene expression in whole blood. PCR products obtained from cDNA of mouse whole blood (+) and -RT controls (−) with primers specific for Tas2r108, -r138, and -r118. GAPDH was amplified as quality control, mouse genomic DNA served as positive control (gD). N, negative control (H_2_O). M - Molecular weight standard (ΦX174 DNA/HaeIII). (E) *In situ* hybridization. Antisense riboprobes specific for α-gustducin and a mixture of riboprobes for Tas2r108, -r119, -r138, -r131 and -r118 in VP sections resulted in specific signals in taste buds. *In situ* hybridization of small intestine sections revealed cells positive for α-gustducin, whereas no signals were detected for Tas2r probes. *In situ* hybridization in colon sections resulted again in the detection of α-gustducin positive cells, but no signals were evident using Tas2r probes. Hybridization with corresponding sense riboprobes (negative control) showed no staining. Scale bars, 100 µm.

To identify cell types expressing Tas2r genes *in vivo*, we examined mouse GI tissues. As a first step, RT-PCR using cDNA prepared from poly(A)^+^ RNA from stomach, duodenum, jejunum, colon and liver of C57BL/6 mice was performed. As expected according to previous studies [24], [25], transcripts of several mouse Tas2rs were detectable (Fig. 1A). The identification of Tas2r108 and -r138 in liver is incongruous with previously published results [25]. All investigated Tas2rs were also detected in mouse VP. We also detected expression of α-gustducin, PLCβ2, TRPM5, Gγ13, Gβ3, and Gβ1 in stomach, duodenum, jejunum, and colon (Fig. 1B) as reported before [3], [7], [26].

To directly compare Tas2r transcript levels in gustatory and non-gustatory tissues we performed quantitative real-time PCR experiments (Fig. 1C). Amplification of Tas2r108, -r118, -r119, -r131, and –r138 from vallate papillae cDNA revealed that all transcripts are present in gustatory tissue, although their relative abundance deviated from each other with Tas2r108 being most highly expressed and Tas2r131 exhibiting the lowest transcript level. The two receptors, Tas2r108 and Tas2r138, showed the most widespread expression in non-gustatory tissues including liver, whereas Tas2r118, -r119, and –r131 mRNA was only detectable in a subset of gastrointestinal tissues. In particular colonic tissue was positive for these 3 receptors, although the abundance of the transcripts was much lower compared to vallate papillae, which is also indicated by the fact that we detected Tas2r118 and Tas2r131 only in one out of three analyzed animals. Obviously, the mRNA level of Tas2r genes in GI tissues is much lower than in vallate papillae and only some Tas2r genes appear to be selectively expressed within the alimentary tract, while others exhibit a more widespread expression pattern.

A possible explanation for the more widespread occurrence of some Tas2r mRNAs might be that the corresponding genes are active in cell types not directly associated with specific tissues or organs such as blood cells. In order to investigate this possibility, we analyzed cDNA from whole blood samples of C57BL/6 mice for the expression Tas2r genes. We selected the receptors Tas2r108 and Tas2r138, two receptors showing a widespread expression in the previous experiment, as well as Tas2r118, a receptor with a very restricted expression pattern. Whereas we did not detect transcripts of Tas2r118 or –r138, a Tas2r108-specific amplicon was evident (Fig. 1D). Thus, the presence of Tas2r108 mRNA in blood cells could indeed account for the apparent widespread expression in a variety of tissues. However, in case of the receptor Tas2r138, the absence of its mRNA in blood cells indicates that other explanations must be taken into consideration. Most importantly, the absence of Tas2r118 mRNA from blood cells correlates well with the observed restricted expression pattern of Tas2r118, Tas2r119, and Tas2r131 suggesting that these genes are indeed predominantly active in colonic tissue.

To visualize Tas2r gene expressing cells *in vivo*, we decided to use the *in situ* hybridization technique well established in our laboratory (e.g. [17], [23], [27], [28]). Hybridization was performed in mouse VP, small intestine and colon sections with probes for α-gustducin or a mixture of Tas2r108, -r138, -r118, -r131, and -r119, to increase the sensitivity of the method. We obtained robust staining using the α-gustducin probe and the Tas2r probe-mixture in taste buds of VP (Fig. 1E). We also observed gustducin-positive epithelial cells in small intestines and colon [3], [4], however, we did not find cells stained with the Tas2r probe mix (Fig. 1E). The specificity of the hybridization procedure was confirmed in parallel experiments using the corresponding sense probes. Next, we investigated the expression of Tas2r138 by immunohistochemical experiments. In contrast to previously reported data [13], immunological detection of this receptor was not possible in mouse GI and taste tissues despite performing these experiments with the same antiserum (see Materials and Methods S1 and Fig. S1).

### qRT-PCR analysis of gastrointestinal tissues in Tas2r131^BLiC^ mice

**Figure 2:**
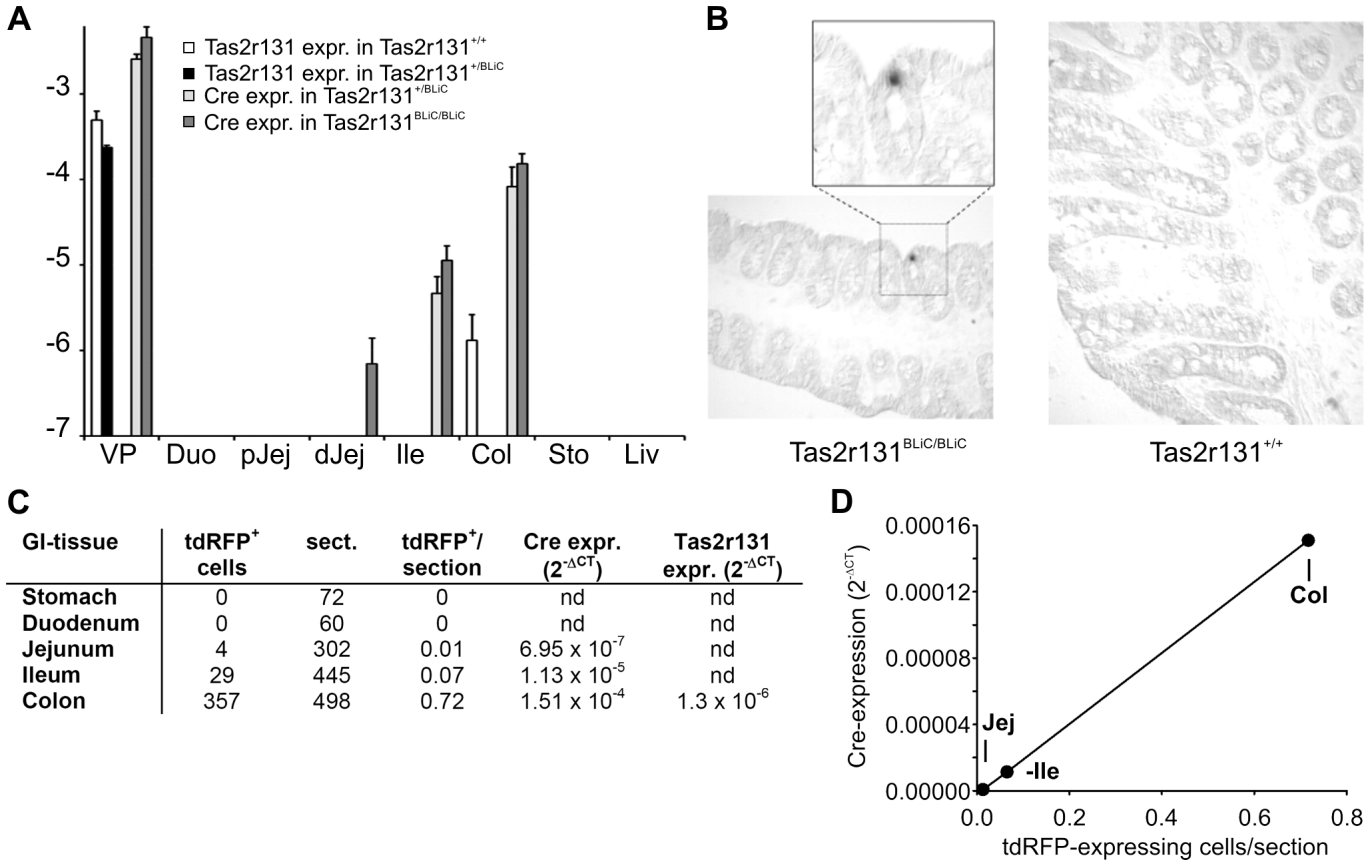
Cre recombinase expression in gastrointestinal tissues of gene-targeted mice. (A) cDNA was prepared from vallate papillae (VP), duodenum (Duo), proximal (pJej) and distal (dJej)jejunum, ileum (Ile), colon (Col), stomach (Sto), and liver (Liv) of homozygous and heterozygous Tas2r131*^BLiC/BLiC^* as well as Tas2r131^+/+^ control mice. The cDNA samples were subjected to quantitative real-time PCR analyses using primers specific for Tas2r131 (white bars, amplification of cDNAs from Tas2r131^+/+^; black bars, amplification of cDNAs from Tas2r131^+/BLiC^; Tas2r131 expression in Tas2r131^BLiC/BLiC^ was not detectable) and Cre-recombinase (dark gray bars, amplification of cDNAs from Tas2r131^BLiC/BLiC^; light gray bars, amplification of cDNAs from Tas2r131^+/BLiC^; Cre expression in Tas2r131^+/+^ was not detectable). Y-axis  =  logarithm of the expression levels relative to β-actin (mean log(2^−ΔCT^) ±SE, n≥3). (B) *In situ* hybridization of colon cross-sections of homozygous Tas2r131^BLiC/BLiC^ mice (left panel) and Tas2r131^+/+^ control mice (right panel). Note that cells expressing Cre-mRNA were only detected in colon sections of Tas2r131^BLiC/BLiC^ mice, but not in the corresponding control tissue. (C) Comparison of the numbers of tdRFP expressing cells in sections of the stomach, duodenum, jejunum, ileum and colon with the expression levels determined by qRT-PCR analyses. The absolute numbers tdRFP expressing cells (tdRFP^+^ cells) and the numbers of sections (sect.) monitored for the various GI-tissues are provided and compared to the values obtained for Cre- and Tas2r131-mRNAs in the corresponding homozygous mouse lines by the qRT-PCR analyses shown in A. (D) Graph showing the correlation between Cre-mRNA expression levels and the number of tdRFP expressing cells per section in distal jejunum (Jej), ileum (Ile) and colon (Col).

To overcome the apparent insufficient sensitivity, we sought to determine the Tas2r expression pattern in the intestines of gene-targeted Tas2r131*^BLiC^* mice. These mice contain an engineered *Tas2r131* allele driving expression of Cre recombinase in Tas2r131-expressing cells. Using quantitative RT-PCR we investigated the occurrence of Tas2r131 and Cre mRNAs in wildtype animals (Tas2r131^+/+^), heterozygous (Tas2r131^+/BLiC^) and homozygous mice (Tas2r131^BLiC/BLiC^) (Fig. 2A). We detected Tas2r131 mRNA in vallate papillae tissue of Tas2r131^+/+^ mice and confirmed the absence of Cre mRNA in these samples as anticipated. Using vallate papillae of Tas2r131^BLiC/BLiC^-mice conversely lead to the detection of Cre-mRNA, but not Tas2r131 mRNA. The amount of Cre-mRNA detected in vallate papillae of Tas2r131^BLiC/BLiC^ mice exceeded the detectable Tas2r131 mRNA in Tas2r131^+/+^-mice by far. Analyses of Tas2r131 and Cre-mRNAs in heterozygous animals, Tas2r131^+/BLiC^, confirmed the relative abundances of the investigated mRNA species, however, compared to the corresponding homozygous animal strains, both mRNA reached only 50% of the levels observed in homozygous animals. In addition to gustatory tissue, we analyzed the expression of Tas2r131 and Cre-mRNA in duodenum, proximal and distal jejunum, ileum, colon, stomach and, as a control, in liver. The most prominent expression was observed in colon tissue. Similar to our qRT-PCR analyses of the expression pattern and level of Tas2rs (Fig. 2A), the detected mRNA levels in colon were much lower than in vallate papillae. Whereas Cre-mRNA was readily detectable in Tas2r131^BLiC/BLiC^ and Tas2r131^+/BLiC^ mice, Tas2r131 mRNA was only barely detectable in Tas2r131^+/+^ colonic tissue. The relative abundance of both mRNA species in Tas2r131^+/+^, Tas2r131^+/BLiC^ and Tas2r131^BLiC/BLiC^ mouse lines paralleled our observations in gustatory tissue, with Cre-mRNA levels in heterozygous animals reaching only 50% of the levels seen in homozygous animals. This reduction by 50% if only one expressing allele is present might have prevented the detection of Tas2r131 mRNA in heterozygous mouse colon tissue. Except for colonic tissue traces of Cre-mRNA were evident in ileum.

This experiment confirmed a proximal to distal gradient in the activity of the Tas2r131 locus within the GI-tract with colon showing the highest activity and suggested that our *in situ* hybridization experiment using Tas2r probes to label bitter receptor expressing cells in the gut may have indeed failed because of the low abundance of Tas2r mRNAs.

### Cre-mRNA detection in colon by in situ hybridization

Our previous experiments indicated that the expression level of Cre-mRNA exceeds by far that of Tas2r131 mRNA. In fact, Cre-mRNA levels detected in colon almost reached the levels of Tas2r131 mRNA in vallate papillae (cf. Figs. 1C and 2A). Hence, we felt that an *in situ* hybridization experiment using a probe to detect Cre-mRNA, which is under the direct control of the Tas2r131 promoter to identify gastrointestinal bitter receptor expressing cells may be more successful than our attempt to directly detect Tas2r mRNAs. Indeed, using a Cre-mRNA-specific antisense probe, we were able to detect a scarce population of Cre-expressing cells (Fig. 2B). The absence of signals in control sections of Cre-negative wildtype animals confirmed the specificity of the experiment.

### Pattern and level of Tas2r131-locus driven expression parallels the number of identified tdRFP-expressing cells

As evident in our qRT-PCR analyses of gastrointestinal tissues in Tas2r131^BLiC^ mice a strong proximal to distal gradient in the activity of the Tas2r131-locus was observed in GI-tissues. Whereas we failed to observe tdRFP expressing cells in sections of the stomach and duodenum and barely detected such cells in sections of the distal jejunum, regular detection was possible in sections of the ileum as well as, most robustly, in colon (Fig.2 C). The frequence of tdRFP expressing cells found in distal jejunum, ileum and colon correlated well with the levels of Cre-mRNA in Tas2r131^BLiC/BLiC^ mice determined by qRT-PCR (Fig. 2A and C) as shown in figure 2D. Also the detection of Tas2r131 mRNA by qRT-PCR of mouse GI-tissues only in the colon of Tas2r131^+/+^ mice is in good agreement with the fact that the highest number of tdRFP expressing cells was observed in this part of the gut.

### Immunohistochemical identification of tdRFP-expressing cells

After confirming activity of the Tas2r131 locus, we analyzed the *in situ* localization of tdRFP-expressing cells. We focused our attention on colon because the RT-PCR analysis indicated a gradient for Cre recombinase expression with the distal parts of the gut showing more robust expression.

Microscopical analyses of tissue sections revealed tdRFP-expressing cells in both the luminal and crypt mucosa. The observed cells can be classified in 2 groups: 1) cells exhibiting a goblet cell-like shape; 2) elongated cells with a columnar shape (Fig. S2). Occasionally, we encountered entire crypts expressing tdRFP. In control sections from wildtype littermates no labeled cells were observed.

**Figure 3:**
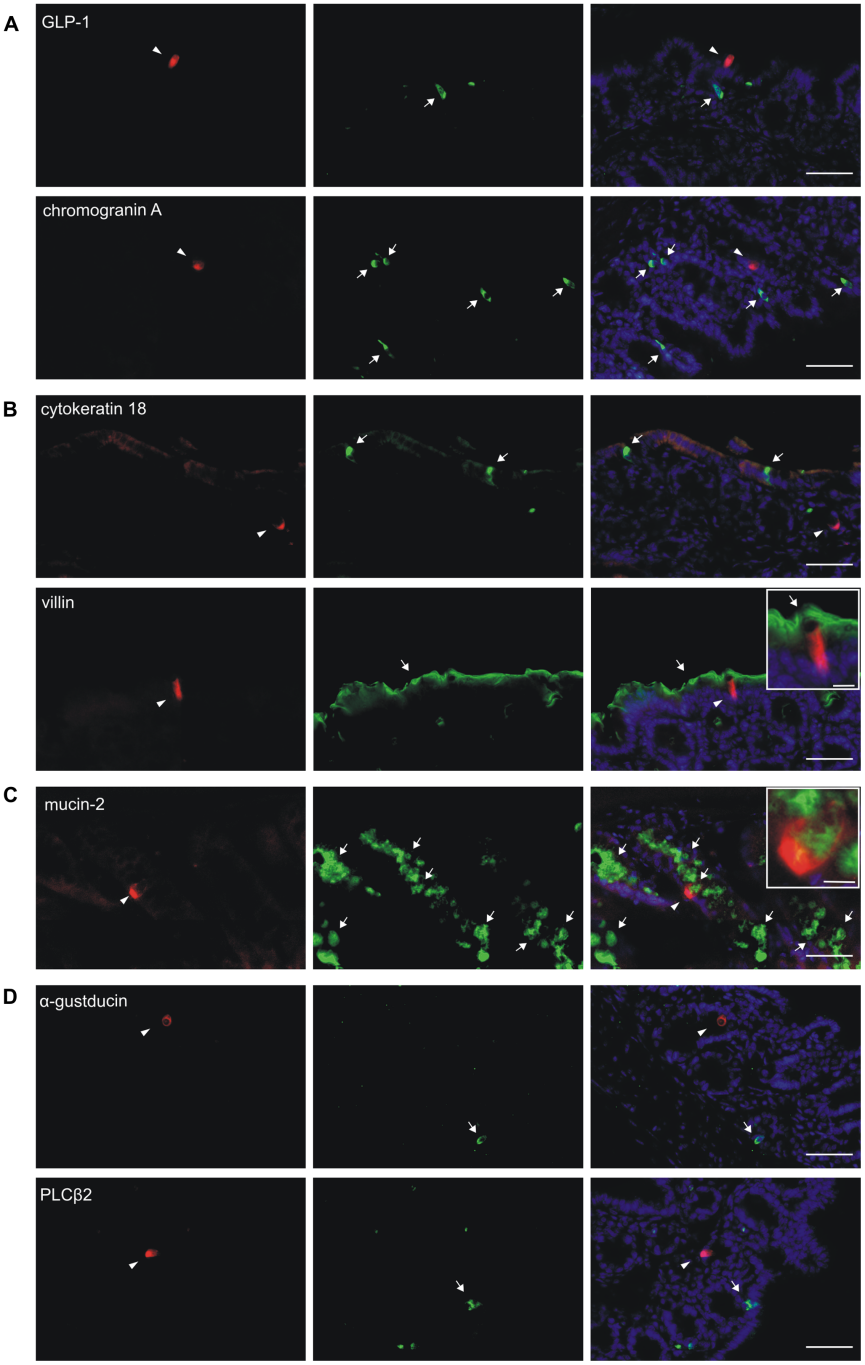
Characterization of tdRFP expressing cells in mouse large intestine. Red tdRFP expressing cells are labeled by arrowheads (left row). Arrows point to cells expressing one of the various marker proteins (green) used in this experiment (middle row). An overlay of the red and green fluorescence is shown in the right row. (A) Labeling of tissue sections with markers of enteroendocrine cells, GLP-1 (upper panel) and chromogranin A (lower panel). (B) Labeling of tissue sections with markers for brush cells and brush border membranes, cytokeratin 18 (upper panel) and villin (lower panel), respectively. (C) Labeling of sections with goblet-cell marker mucin-2. (D) Labeling of tissue sections with markers of taste-like cells, α-gustducin (upper panel) and PLCβ2 (lower panel). Scale bars, 50 µm; 10 µm (insets).

To establish the types of colonic cells expressing Tas2r131 as indicated by tdRFP fluorescence, we conducted immunohistochemical experiments. For that purpose, we used antisera specific for marker proteins allowing the classification of main epithelial cell types present in the gut: GLP-1, a marker for enteroendocrine L-cells cells [2], was shown to co-localize with sweet taste receptor subunits and taste signalling proteins in mammalian gut [3], [7], [12]. As a general marker for enteroendocrine cells, we chose chromogranin A [2], which was demonstrated to co-label α-gustducin expressing cells in human colon and mouse small intestine [4], [12]. As shown in Fig. 3A, neither GLP-1, nor chromogranin A was detected in tdRFP-expressing colonic cells of our gene-targeted mice.

Intestinal brush cells express Tas1r3 as well as α-gustducin and other taste signalling proteins [5], [6], [26], [29], and their presence can be visualized using cytokeratin 18 as marker [6]. Again, no co-localization of tdRFP signals with cytokeratin 18-positive cells was evident (Fig. 3B). Colonocytes express villin in their luminal membrane [30]. However, a villin-specific antiserum (Fig. 3B) revealed no co-localization with tdRFP signals, as the luminal membrane of tdRFP expressing cells seems not to possess a brush border (Fig. 3B inset).

As marker for goblet cells we chose mucin-2, an abundant protein produced by intestinal goblet cells [31]. Before being released, mucin-2 is stored in granules that are in mature goblet cells situated in the “teca”, an apical cup-like structure made of microtubules and intermediate filaments [32]. Our experiments demonstrated that mucin-2 occasionally colocalizes with tdRFP (Fig. 3C). The inset shows that mucin-2 immunoreactivity is confined to a cup-like cell structure surrounded by a thin cytoplasmic layer resembling intestinal goblet cells.

Additionally, we stained colon sections with antisera against α-gustducin and PLCβ2. Neither of these taste cell markers colocalized with tdRFP signals (Fig. 3D). For each antibody used, the corresponding negative controls did not result in signals, indicating the specificity of staining procedure (Fig. S3).

## Discussion

In the present study we investigated the localization and putative role of Tas2rs expressed in the GI tract. In the course of our experiments, we encountered considerable difficulties to identify cells expressing Tas2rs *in vivo*. This perhaps explains, why a decade after the first report about Tas2r expression in the gut [24], evidence on cell types expressing Tas2rs *in situ* is scarce, compared to the better investigated expression of Tas1r genes. On the one hand this is explained by a considerable lack of experimental tools for the investigation of Tas2rs, which are readily available for Tas1r research. These tools include sufficiently specific antisera such as for the sweet taste receptor subunits Tas1r2 (e.g. [4], [33]) and Tas1r3 (e.g. [4], [5], [33]), mouse models genetically modified in loci corresponding to Tas1r genes (e.g. [34]), which are scarce for Tas2rs as evident from our attempts to label Tas2r138 expressing cells with a specific antiserum. On the other hand a particular problem for the investigation of bitter taste receptor function in the GI tract is a lack of functional data for the majority of mouse Tas2rs, which makes it more difficult correlating specific stimuli with physiological responses in experimentally accessible animals (cf. [1]). This is again in sharp contrast to elegant physiological experiments that were possible in mouse studying gastrointestinal mouse sweet taste receptor function (e.g. [3], [4], [35]), because this receptors has previous been extensively characterized *in vitro*.

In good agreement with previous reports the expression of several *Tas2r* genes in gastrointestinal tissues of mice was evident by RT-PCR-analysis (Fig. 1). However, the relative abundance of Tas2r transcripts in gustatory tissue and GI tissues deviated from each other considerably. The mRNA levels of all analyzed Tas2rs in gustatory tissues exceeded by far the levels of the corresponding mRNAs in GI tissues. Moreover, it appears that a subset of Tas2r genes investigated by RT-PCR in GI tissue exhibits a more restricted expression pattern and that particularly these most specifically expressed transcripts are even less abundant. This can be explained in part by a lack of expression of such Tas2r genes in blood cells (Fig. 1D) or other cell types not restricted to specific tissues or organs. Moreover, the finding of expression of some Tas2r genes in blood cells indicates that great care has to be taken when interpreting the results of tissue-specific RT-PCR experiments for bitter receptors.

When analyzing the genetically modified mouse strain, Tas2r131^BLiC^, by qRT-PCR it became apparent that amplification of Cre-mRNA resulted in higher expression values than analyzing equivalent tissue samples of wildtype control mice for Tas2r131 mRNA. If one assumes that the transcriptional activity is similar for both coding regions as they are transcribed from the same gene locus, this inconsistency may reflect different mRNA stabilities of the transcripts, perhaps caused by the Cre sequence possessing a stabilizing BGH polyadenylation signal [36]. Moreover, the fact that heterozygous Tas2r131*^+/BLiC^* mice showed roughly 50% of the transcript levels for both mRNA species indicates a linear relationship between the number of active gene loci and mRNA abundance and hence, suggests that no other confounding factors influence cDNA detection. Most importantly, in the two tissues exhibiting elevated Cre-mRNA levels, vallate papillae and colon, Tas2r131 mRNA has been detectable allowing to conclude that Cre expression is truly reporting activity of the Tas2r131 gene locus (Fig. 2A). This notion is further supported by our *in situ* hybridization experiment using a Cre-specific probe to detect cells actively expressing this mRNA in colon sections (Fig. 2B).

Despite the obviously low expression levels of Tas2r genes, by using gene-targeted Tas2r131*^BLiC^*/ROSA26^tdRFP^ mice, we identified epithelial cells in the distal part of the GI tract, which are positive for the reporter protein indicating Tas2r131 gene expression. The present finding is an important contribution to elucidating the role of Tas2rs in the gut and may help to link physiological responses elicited by bitter substances in the digestive tract [11], [15], [16] and the spatially undefined presence of the Tas2r mRNAs detected by RT-PCR analyses.

Several studies in model cell lines (e.g. [10], [12], [14]) as well as a single histochemical study using an antibody raised against mouse Tas2r138 [13] point to enteroendocrine cells as putative bitter sensing cells. We could not confirm this observation for Tas2r131, as there was no colocalization of tdRFP with enteroendocrine cell marker chromogranin A [12] or L-type cell-specific gut hormone GLP-1 [3]. Intestinal brush cells are also frequently mentioned as candidates for chemosensing cells in the GI tract [5]–[7], [26]. As we did not find colocalization of tdRFP fluorescence with brush cell marker cytokeratin-18 [6] or villin [30], we conclude that this cell type may not contribute to bitter compound recognition by Tas2r131 (Fig. 3). Interestingly, tdRFP-positive cells were also not labeled with α-gustducin and PLCβ2 (Fig. 3), widely used markers for taste-like cells (e.g. [3], [7], [12], [29]). A possible explanation for this might be the existence of alternative effector proteins, such as other Gα-subunits (e.g. [37]).

Using antibodies against mucin-2, we identified a number of tdRFP-positive cells as goblet cells (Fig. 3). Goblet cells produce mucus, a protective layer of glycoproteins and other molecules covering the intestinal epithelium [31],[32]. Tas2r presence in these cells may indicate a defense-related function of the receptors, possibly involving the recognition of harmful xenobiotics. A similar role was suggested in a study showing that bitter substances increase anion transport and fluid secretion in the colon [11]. Moreover, goblet cells are present along small and large intestine, but their number increases, similar to the expression gradient we observed for Tas2r131, in the distal parts of the gut, where the concentration of bacteria is the highest [32]. Since another recent report suggests that Tas2rs in the nasal epithelium recognize bacterial quorum sensing signals [38], one may speculate that intestinal Tas2rs fulfill a role in gut protection against bacterial pathogens.

Goblet cells constitute up to 16% of epithelial cells in mouse colon [31]; however, we observed tdRFP fluorescence only in a very small subset. Since our mouse strain represents only one of over 30 functional Tas2rs [8] and bitter taste receptor cells express only subsets of Tas2rs [23], other Tas2rs may be expressed in separate or partially overlapping populations of goblet cells similar to the situation in human bitter taste receptor cells [23].

Apart from the goblet cell population, a second type of epithelial cells of a columnar shape displayed tdRFP fluorescence (Fig. S2). Unfortunately, none of the antibodies used in this study labeled this cell population. Since the goblet cell-directed antibody specifically labels the mucus-containing cup-like structures and not the cytoplasm, those unidentified cells may in fact be goblet cells devoid of their “cups” due to the sectioning of the tissue. However, those cells may also represent a population of stem cells or immature cells not sharing the marker proteins of differentiated cell types [39]. This hypothesis is supported by the fact that we occasionally encountered entire crypts showing tdRFP fluorescence, perhaps originating from isolated Tas2r131-positive stem cells. However, the exact origin of the tdRFP-positive crypts and characteristics of the so far unidentified cells needs to be carefully investigated.

In conclusion, the use of Tas2r131*^BLiC^*/ROSA26^tdRFP^ mice enabled us to demonstrate Tas2r131 expression in a subpopulation of colonic cells, partially identified as goblet cells. Future studies employing this mouse line will reveal molecular and physiological characteristics of intestinal bitter-sensing cells and help to associate the presence of Tas2rs in the GI tract with their function.

## Supporting Information

Figure S1 **Immunodetection of mouse Tas2r138.** Since a recent report suggested that Tas2r138 can be detected by immunohistochemistry in mouse small intestine [13], we took advantage of this new possibility to identify bitter receptor expressing cells. Immunohistochemical staining was performed on sections of VP and small intestines. To confirm staining specificity, antibody preabsorption with immunogenic peptide was included. (A) Top: PLCβ2 (red) but not Tas2r138 (green) can be detected in taste bud cells of VP and in (B) small intestines. Bottom: antibody preabsorbed with blocking peptide (negative control). Scale bars, 75 µm. (C) Immunodetection of Tas2r138 overexpressed in HEK 293 cells. HEK-293 cells were transfected with SST-Tas2r138-HSV-expressing or empty pcDNA5/FRT vector. 24 h after transfection the cells were fixed and double staining with antibodies against Tas2r138 and against HSV was performed. Top panel: the cells expressing the HSV-tagged Tas2r138 receptor are labelled with anti-HSV (red) but not anti-Tas2r138 (green) antibody. Bottom panel: no staining was visible in cells transfected with an empty vector. Scale bar: 50 µm.
(TIF)

Figure S2 **Different morphological types of tdRFP expressing cells.** (A) tdRFP expressing cell with goblet cell morphology in the mouth of a crypt. (B) tdRFP expressing cells with colonocyte morphology. Images were obtained from 14 µm sections of mouse colon. Scale bar: 20 µm.
(TIF)

Figure S3 **Control immunohistochemical reactions did not reveal antibody signals.** (A, upper panel) GLP-1, (D, upper panel) α-gustducin, and (B, lower panel) villin staining specificity was demonstrated by preabsorbing the corresponding antibody with an excess of immunogenic peptide. (D, lower panel) PLCβ2, (C) mucin-2, (A, lower panel) chromogranin A and (B, upper panel) cytokeratin 18 staining specificity was demonstrated by omitting the primary antibody during overnight incubation. All shown immunohistochemical stainings were performed on 14 µm sections of mouse colon. Scale bars: 50 µm.
(TIF)

Materials and Methods S1 **Immunocytochemical and immunohistochemical staining procedures.**
(DOC)

Table S1 **List of primers used for analytical RT-PCR including sequence, annealing temperature, and product size.**
(DOCX)

Table S2 **List of oligonucleotides used for quantitative RT-PCR.**
(DOCX)
