# Supplementary figures and images for: A Subset of Mouse Colonic Goblet Cells Expresses the Bitter Taste Receptor Tas2r131
Source: PLoS One. 2013 Dec 18;8(12):e82820. doi: 10.1371/journal.pone.0082820 (PMC3867391; doi:10.1371/journal.pone.0082820)

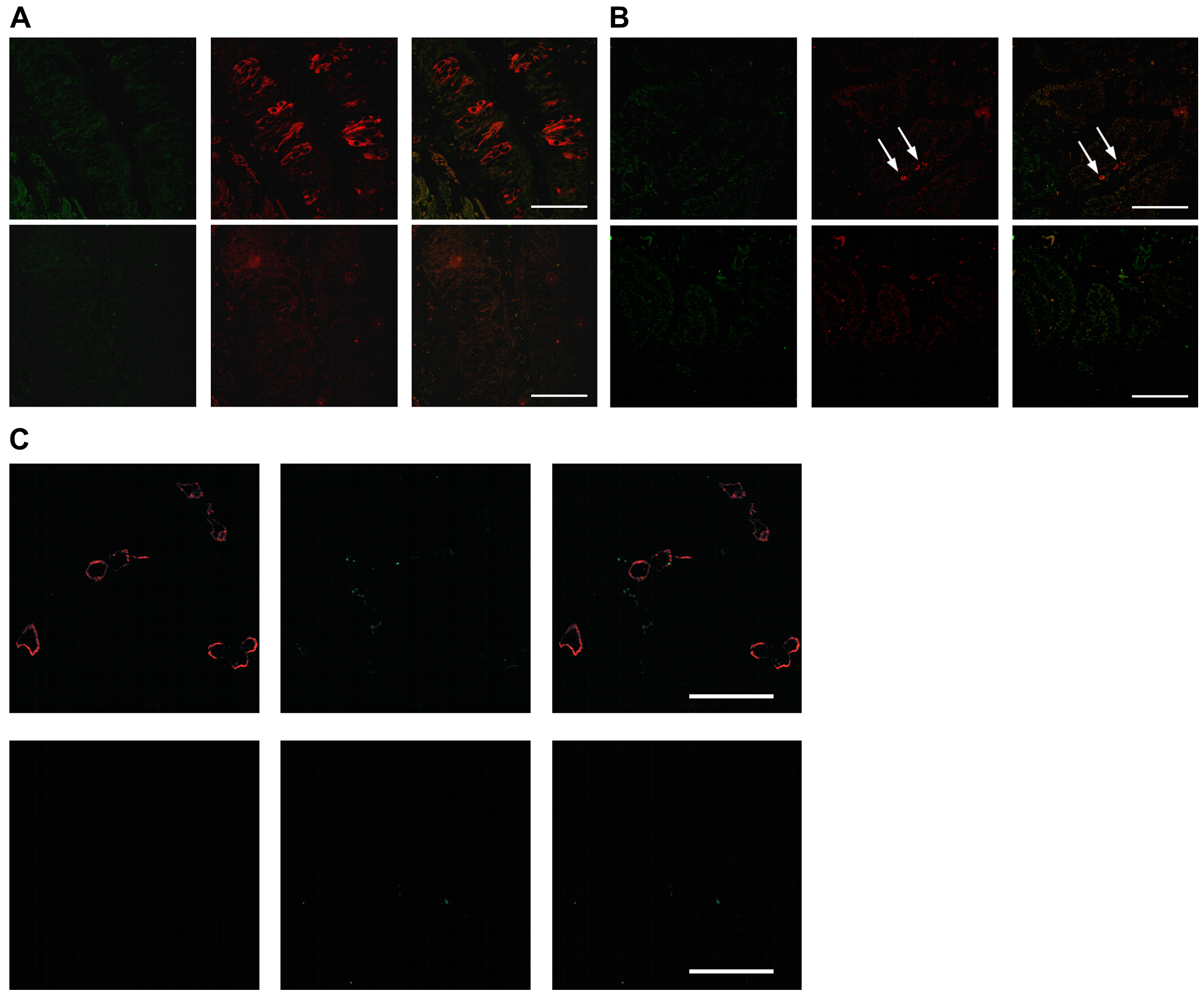

Supplement: Figure S1 — Immunodetection of mouse Tas2r138. Since a recent report suggested that Tas2r138 can be detected by immunohistochemistry in mouse small intestine [13], we took advantage of this new possibility to identify bitter receptor expressing cells. Immunohistochemical staining was performed on sections of VP and small intestines. To confirm staining specificity, antibody preabsorption with immunogenic peptide was included. (A) Top: PLCβ2 (red) but not Tas2r138 (green) can be detected in taste bud cells of VP and in (B) small intestines. Bottom: antibody preabsorbed with blocking peptide (negative control). Scale bars, 75 µm. (C) Immunodetection of Tas2r138 overexpressed in HEK 293 cells. HEK-293 cells were transfected with SST-Tas2r138-HSV-expressing or empty pcDNA5/FRT vector. 24 h after transfection the cells were fixed and double staining with antibodies against Tas2r138 and against HSV was performed. Top panel: the cells expressing the HSV-tagged Tas2r138 receptor are labelled with anti-HSV (red) but not anti-Tas2r138 (green) antibody. Bottom panel: no staining was visible in cells transfected with an empty vector. Scale bar: 50 µm. (TIF) [file pone.0082820.s001.tif]

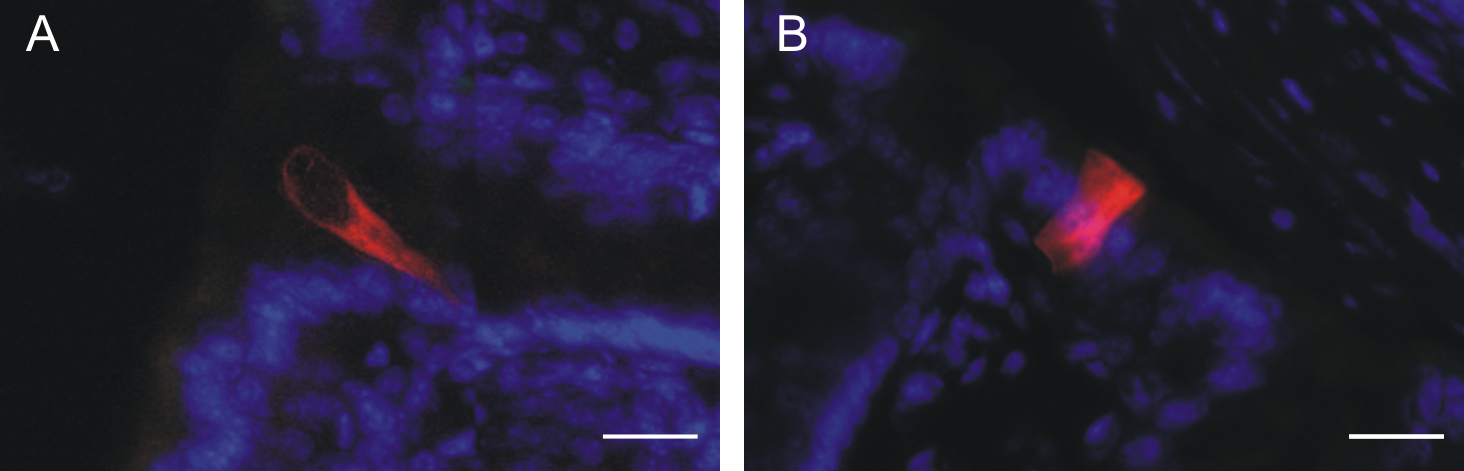

Supplement: Figure S2 — Different morphological types of tdRFP expressing cells. (A) tdRFP expressing cell with goblet cell morphology in the mouth of a crypt. (B) tdRFP expressing cells with colonocyte morphology. Images were obtained from 14 µm sections of mouse colon. Scale bar: 20 µm. (TIF) [file pone.0082820.s002.tif]

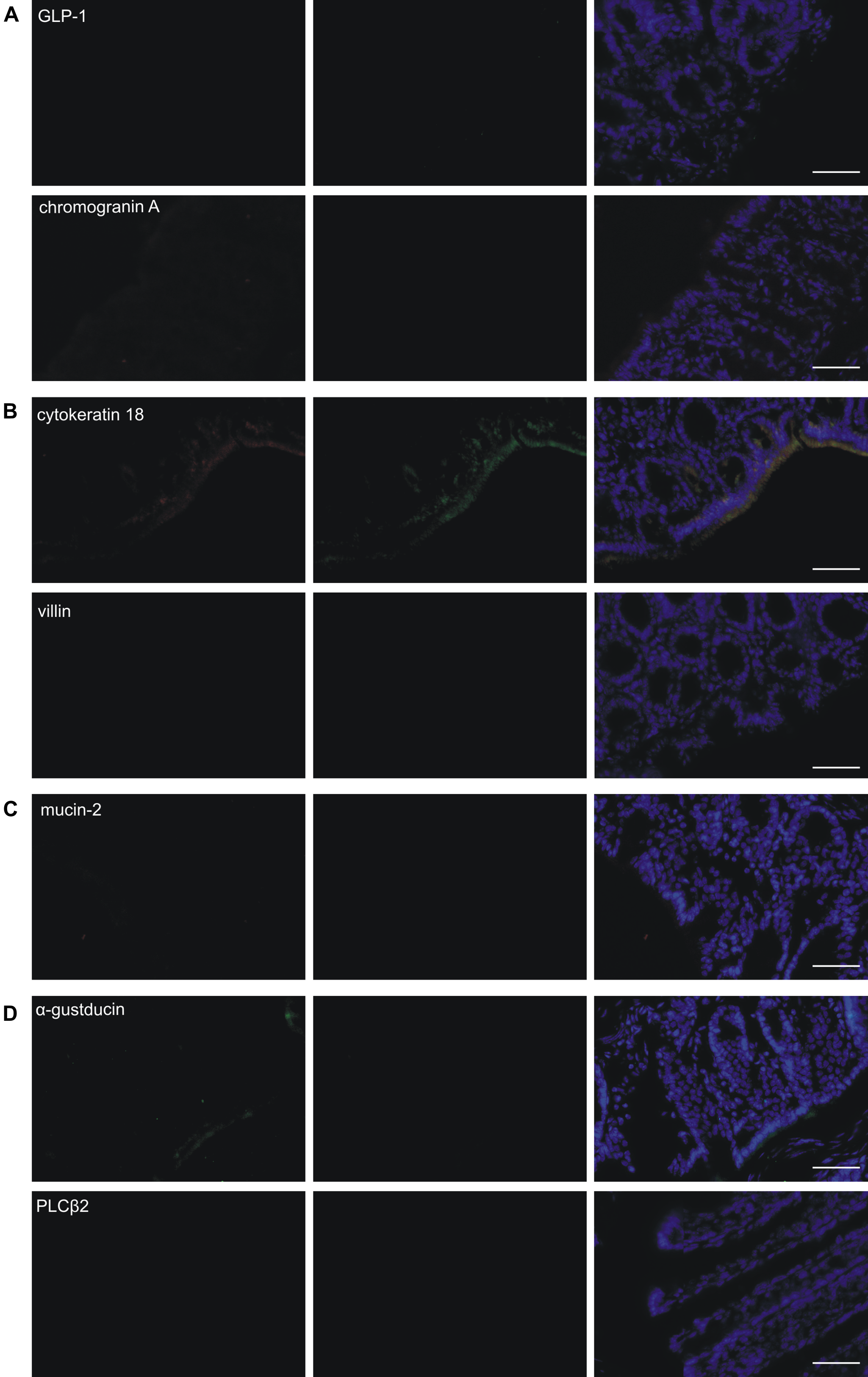

Supplement: Figure S3 — Control immunohistochemical reactions did not reveal antibody signals. (A, upper panel) GLP-1, (D, upper panel) α-gustducin, and (B, lower panel) villin staining specificity was demonstrated by preabsorbing the corresponding antibody with an excess of immunogenic peptide. (D, lower panel) PLCβ2, (C) mucin-2, (A, lower panel) chromogranin A and (B, upper panel) cytokeratin 18 staining specificity was demonstrated by omitting the primary antibody during overnight incubation. All shown immunohistochemical stainings were performed on 14 µm sections of mouse colon. Scale bars: 50 µm. (TIF) [file pone.0082820.s003.tif]
