# Supplementary material for: A Subset of Mouse Colonic Goblet Cells Expresses the Bitter Taste Receptor Tas2r131
Source: PLoS One. 2013 Dec 18;8(12):e82820. doi: 10.1371/journal.pone.0082820 (PMC3867391; doi:10.1371/journal.pone.0082820)
Supplement: Materials and Methods S1 — Immunocytochemical and immunohistochemical staining procedures. (DOC) [file pone.0082820.s004.doc]

**Materials and Methods S1**

*Immunocytochemistry-* HEK 293 cells were maintained at 37°C, 5% CO_2_ and 95% humidity in adherent culture in high-glucose DMEM (Invitrogen) supplemented as above. Transfection of HEK 293 cells was performed as described before [1,2]. Briefly, cells were transiently transfected with pcDNA5/FRT vector (Invitrogen) containing a construct coding for the mouse bitter taste receptor Tas2r138 tagged with the first 45 amino acids of rat somatostatin type 3 receptor at the N-terminus and with *Herpes simplex* virus (HSV) glycoprotein D epitope at the C-terminus. 24 hours after transfection immunostaining with anti-HSV and anti-Tas2r138 antibodies was performed. As a control for labelling specificity, preabsorption of the anti-Tas2r138 antibody with the immunogenic peptide was performed. HSV epitope was detected with mouse monoclonal antibodies (1:15000, Novagen) and anti-mouse antibodies conjugated with Cy3 (1:2000, Sigma). Tas2r138 was detected with goat polyclonal antibodies (1:100, sc-34357, Santa Cruz Biotechnology, Heidelberg, Germany) and anti-goat antibodies conjugated with Alexa 488 (1:2000, Molecular Probes). Images were taken with a confocal laser scanning microscope (Leica TCS SP2). Alexa 488 was excited at 488 nm and detected at 510-560 nm. Cy3 was excited at 561 nm and detected at 570-600 nm.

*Immunohistochemistry*- For the detection of Tas2r138, paraffin sections of C57BL/6 mouse VP and small intestine were dewaxed for 2 min and 5 min in toluene and rehydrated in a graded ethanol series (5 min 100%, 3 min 100%, then 96%, 70%,  40%, 2 min each) followed by 3 x 2 min washes in deionized water. For antigen retrieval sections were heated to 95°C in 10 mM sodium citrate buffer, pH 6.0 (3 x 5 min). After cooling, sections were rinsed 2 x 3 min in deionized water, then 5 min in PBS and incubated for 1 h in blocking solution (10% normal horse serum, 0.2% Triton X-100, and 1% BSA in PBS) and then overnight at 4°C with primary antibodies in PBS containing 3% normal horse serum, 0.2% Triton X-100, and 1% BSA. Subsequently, sections were washed 3 x 15 min in PBS, followed by a 1 h incubation with secondary antibodies and 3 washes in PBS, for 15 min each. Sections were mounted with Fluorescent Mounting Medium (Dako) and analyzed by confocal microscopy (Leica TCS SP2) using 488 nm excitation wavelength and 500-530 nm detection wavelengths.

For detecting α-gustducin, PLCβ2, GLP-1, chromogranin A, villin, and mucin-2 frozen sections of Tas2r131*^BLiC^*/ROSA26-tdRFP large intestine were fixed in 4% paraformaldehyde in PBS, and then rinsed 3 times in PBS. An antigen retrieval step (10 min, 80°C, 10 mM sodium citrate, pH 6) was performed for chromogranin A, villin and mucin-2 staining. After cooling, sections were washed 3 times in PBS, and incubated with blocking solution (5% normal horse serum, 0.5% Triton X-100 in PBS), followed by overnight incubation at 4°C with primary antibodies diluted in PBS containing 5% normal horse serum and 0.2% Triton X-100. Next, sections were washed 3 times in PBS, and secondary antibodies were applied for 1 h, followed by 3 washes in PBS. Sections were counter-stained with DAPI, washed twice in PBS and mounted as above.

For detecting cytokeratin 18, a modified version of the protocol published by Hass et al. was used [3]. Briefly, frozen sections of Tas2r131*^BLiC^*/ROSA26-tdRFP large intestine were fixed in ice-cold acetone for 8 min, then rinsed 3 times in PBS and incubated in citrate buffer (10 mM sodium citrate, pH 6, 0.3% Tween-20) at 80 °C for 10 min. After washing 3 times in PBS, sections were blocked with 10% normal goat serum in PBS, and incubated overnight with a biotin-conjugated anti-cytokeratin 18 antibody in PBS containing 10% normal goat serum and 0.3% Triton X-100. Next, the sections were washed 3 times in PBS, and streptavidin was applied for 20 min, followed by 3 washes in PBS. Counter staining with DAPI and mounting was done as described above.

Tas2r138 was labeled using a goat polyclonal antiserum (1:100, sc-37354, Santa Cruz Biotechnology); α-gustducin with a rabbit polyclonal antiserum (1:400, sc-395, Santa Cruz Biotechnology); PLCβ2 with a rabbit polyclonal antiserum (1:5000, sc-206, Santa Cruz Biotechnology); GLP-1 with a goat polyclonal antiserum (1:1000, sc-7782, Santa Cruz Biotechnology); mucin-2 with a rabbit polyclonal antiserum (1:50, sc-15334, Santa Cruz Biotechnology); chromogranin A with a rabbit polyclonal antiserum (1:1000, 20086, Immunostar); cytokeratin 18 with mouse monoclonal biotin-conjugated antibody (1:10, 61528, Progen Biotechnik); villin with goat polyclonal antiserum (1:200, sc-7672, Santa Cruz Biotechnology).

Specificity of the labeling for Tas2r138, α-gustducin, GLP-1 and villin was verified by preabsorbing the primary antibody with the corresponding blocking peptides. Specificities of PLCβ2 and mucin-2 antibodies were verified previously by incubation with tissue sections from PLCβ2 and mucin-2 knockout mice [4,5], and by omitting primary antibodies during incubation of control sections. Specificity of chromogranin A and cytokeratin 18 labeling was verified by incubating control tissue sections without primary antibodies. Primary antibodies were visualized with appropriate anti-rabbit or anti-goat secondary antibodies (1:2000) or streptavidin (1:800) conjugated with AlexaFluo488 (Molecular Probes).

Sections were analyzed either by using a MIRAX MIDI system with filter sets for FITC, Cy3 and DAPI (Zeiss) or a fluorescence microscope with filter sets for FITC, Cy3 and DAPI (Axioplan, Zeiss) equipped with a CCD camera (RT slider, Diagnostic Instrument). Digital images were analyzed using Mirax Viewer (Zeiss) or Metamorph software (Visitron Systems GmbH). Pictures were modified for brightness and contrast, and assembled using CorelDRAW Graphic Suite.

1. Behrens M, Brockhoff A, Kuhn C, Bufe B, Winnig M et al. (2004) The human taste receptor hTAS2R14 responds to a variety of different bitter compounds. Biochem Biophys Res Commun 319: 479-485.

2. Bufe B, Hofmann T, Krautwurst D, Raguse JD, Meyerhof W (2002) The human TAS2R16 receptor mediates bitter taste in response to beta-glucopyranosides. Nat Genet 32: 397-401.

3. Hass N, Schwarzenbacher K, Breer H (2007) A cluster of gustducin-expressing cells in the mouse stomach associated with two distinct populations of enteroendocrine cells. Histochem Cell Biol 128: 457-471.

4. Van der Sluis M, De Koning BA, De Bruijn AC, Velcich A, Meijerink JP et al. (2006) Muc2-deficient mice spontaneously develop colitis, indicating that MUC2 is critical for colonic protection. Gastroenterology 131: 117-129.

5. Kim JW, Roberts C, Maruyama Y, Berg S, Roper S et al. (2006) Faithful expression of GFP from the PLCbeta2 promoter in a functional class of taste receptor cells. Chem Senses 31: 213-219.
