# Supplementary material for: A Subset of Mouse Colonic Goblet Cells Expresses the Bitter Taste Receptor Tas2r131
Source: PLoS One. 2013 Dec 18;8(12):e82820. doi: 10.1371/journal.pone.0082820 (PMC3867391; doi:10.1371/journal.pone.0082820)
Supplement: Table S1 — List of primers used for analytical RT-PCR including sequence, annealing temperature, and product size. (DOCX) [file pone.0082820.s005.docx]

Table S1. List of primers used for analytical RT-PCR including sequence, annealing temperature, and product size.

| Primer | Sequence 5’ – 3’ | Annealing temp. | Product size |
| --- | --- | --- | --- |
| **RT-PCR** |  |  |  |
| gustducin for | GTTTGAGCAAATCAACTGCCC | 58°C | 733 bp |
| gustducin rev | TCATGCATTCTGTTCACCTCC |  |  |
| PLCβ2 for | AAAGAAGTGACAGAGCCACAG | 58°C | 716 bp |
| PLCβ2 rev | TTCTCCTGGAACTGCTTTTCC |  |  |
| TRPM5 for | CAGGGCTTCTTCACAGATGAG | 58°C | 778 bp |
| TRPM5 rev | GCAAGTCTCTCTCCAGATGTTG |  |  |
| Gγ13 for | CTGTCTCCTCCAAAACCTCAG | 58°C | 281 bp |
| Gγ13 rev | CACACCTTACAGAGAGTGTGG |  |  |
| Gβ1 for | AGATGAGTGAACTTGACCAGC | 58°C | 721 bp |
| Gβ1 rev | GCATTGCCATTGGGAAAGAAAC |  |  |
| Gβ3 for | ACATCTGGCCAAGATCTATG | 58°C | 635 bp |
| Gβ3 rev | AATAGGCAGTCAGTTCCTGG |  |  |
| Tas2r108 for | GGTCAACAGTCGCAGAATTGC | 61°C | 717 bp |
| Tas2r108 rev | TGTCCTGGAGGGTAAGCAGC |  |  |
| Tas2r118 for | AACGCAAGTCACCATCTTCTC | 58°C | 800 bp |
| Tas2r118 rev | AACCATAGATGACAGCTTCGC |  |  |
| Tas2r119 for | ATCCTGTTTGGTGAGACACAC | 58°C | 767 bp |
| Tas2r119 rev | TCTTGTTGGCTGAGTGATGAG |  |  |
| Tas2r138 for | CGCTTCTCTCACACCTTTCC | 58°C | 421 bp |
| Tas2r138 rev | AAGCAGCACAGAATGACACC |  |  |
| Tas2r131 for | GACTGGAGAATCTATGAAGGGAGAGGATCAG | 65°C | 508 bp |
| Tas2r131 rev | CTGCTTGAAACACTTCCTATTTGGGGAG |  |  |
|  |  |  |  |
| GAPDH for | ACCACAGTCCATGCCATCAC | 58°C | 453 bp |
| GAPDH rev | TCCCACCACCCTGTTGCTGTA |  |  |
