# Supplementary material for: A Subset of Mouse Colonic Goblet Cells Expresses the Bitter Taste Receptor Tas2r131
Source: PLoS One. 2013 Dec 18;8(12):e82820. doi: 10.1371/journal.pone.0082820 (PMC3867391; doi:10.1371/journal.pone.0082820)
Supplement: Table S2 — List of oligonucleotides used for quantitative RT-PCR. (DOCX) [file pone.0082820.s006.docx]

**Table S2.** List of oligonucleotides used for quantitative RT-PCR.

| Assay | Primer | Sequence 5’ – 3’ |
| --- | --- | --- |
| Tas2r108 | Tas2r108 probe | FAM-CAGACGGAGGCTCACATGGGTGC-TAM |
|  | Tas2r108 for | AACAGGACCAGCTTTTGGAATC |
|  | Tas2r108 rev | GAGGAAACAGATCATCAGCCTCAT |
| Tas2r118 | Tas2r118 probe | FAM-TCAGCCTGGGCATCTCACATTTCTGTC-TAM |
|  | Tas2r118 for | CACCGGTGGAGACGATTCTC |
|  | Tas2r118 rev | AAGTTGTATAGCATTGATGTCCACTGT |
| Tas2r119 | Tas2r119 probe | FAM-AACCCAAGACTCAGTGACTTGCCAGTGC-TAM |
|  | Tas2r119 for | CAAGAGCTTGGGTCACCTCAA |
|  | Tas2r119 rev | TGTTGGCTGAGTGATGAGTAGCA |
| Tas2r131 | Tas2r131 probe | FAM-TAGCCCACATTTCCCATCCCCTTTTC-TAM |
|  | Tas2r131 for | CTGCCTGAGCATATTCTACTTATTCAA |
|  | Tas2r131 rev | CACCTCTCAATCTCCACTTAAACCA |
| Tas2r138 | Tas2r138 probe | FAM-CCTGCAGGGCCTTCTGCTTCTGG-TAM |
|  | Tas2r138 for | GTCTCAGCATCACTCGGCTTT |
|  | Tas2r138 rev | GCAGGCGAGCTGAATAGCA |
| Cre-recombinase | Cre probe | FAM-CACCAGCCAGCTATCAACTCGCGC-TAM |
|  | Cre for | TTGCCGCGCCATCTG |
|  | Cre rev | TTGCTTCAAAAATCCCTTCCA |
| β-actin | β-actin probe | FAM-TTGAGACCTTCAACACCCCAGCCA-TAM |
|  | β-actin for | TACGACCAGAGGCATACAG |
|  | β-actin rev | GCCAACCGTGAAAAGATGAC |
